# *TP53* mutations predict poor response to immunotherapy in patients with metastatic solid tumors

**DOI:** 10.1002/cam4.5953

**Published:** 2023-04-20

**Authors:** Ji‐Yeon Kim, Jaeyun Jung, Kyoung‐Mee Kim, Jeeyun Lee, Young‐Hyuck Im

**Affiliations:** ^1^ Division of Hematology‐Oncology, Department of Medicine, Samsung Medical Center Sungkyunkwan University School of Medicine Seoul South Korea; ^2^ Innovative Institute for Precision Medicine, Samsung Medical Center Seoul South Korea; ^3^ Department of Pathology and Translational Genomics, Samsung Medical Center Sungkyunkwan University School of Medicine Seoul South Korea

**Keywords:** chemotherapy, gene expression, immunotherapy, metastatic cancer, *TP53* mutation

## Abstract

**Background:**

TP53 is the most commonly mutated gene across all cancer types. R175H mutation was considered structural mutation where the mutation causes misfolding of the protein and leads to a significant conformational alterations within p53's DNA binding domain. The aim of this study was to explain the reason why R175H worse the response to immunotherapy by analyzing tumor immune microenvironment through the expression of immune cells and PD‐1.

**Materials and Methods:**

Patients diagnosed with metastatic carcinoma, including colorectal cancer (CRC), breast cancer (BRCA), gastric cancer (GC), non‐small cell lung cancer (NSCLC), and 20 other cancer types, treated in a palliative setting at Samsung Medical Center between October 2019 and April 2021, were enrolled. Of these patients, those who underwent TDS analysis (TruSight™ Oncology 500 assay [TSO 500]) were finally analyzed.

**Results:**

Of 1770 patients, 1012 (57.2%) harbored genetic alterations in TP53. All mutations were single nucleotide variants (SNVs), and the most frequent SNV was R175H (*n* = 84, 7.5%) which was known as one of the most common hotspot TP53 mutation. The overall survival of patients with TP53 R175H mutations was significantly worse following chemotherapy (606 vs. 456 days, *p* < 0.001) or immunotherapy (822 vs. 350 days, *p*  < 0.001) compared to those with TP53 mutation in other loci. RNA sequencing indicated that the immune response‐related pathways were downregulated in tumors harboring TP53 R175H mutation. Moreover, the expression of CD8(+) T cells PD‐1 were lowered in R175H mutation tumors. In the analysis of TP53 structural domain, compared to those having TP53 mutation in other domain, patients with mutations occurring in the nuclear exporter signal (NES) and E4F1‐binding domains had significantly worse overall survival following chemotherapy (NES: 606 vs. 451 days, *p* = 0.043; E4F1: 606 vs. 469 days, *p* = 0.046) and immunotherapy (NES: 822 vs. 403 days, *p*  < 0.001; E4F1: 822 vs. 413 days, *p*  < 0.001). In addition, tumors with TP53 mutation and co‐existing copy number amplification of CCND1, FGF4, and FGF19 in chromosome 11 conferred worse prognosis than those with only TP53 mutation (*p*  < 0.050).

**Discussion:**

Each TP53 mutations indicated differential treatment outcomes following chemotherapy or immunotherapy in patients with metastatic cancer. Functional analysis including RNASeq suggested that TP53 mutation downregulated immune response.

**Conclusion:**

Overall, we found each *TP53* mutation to indicate different prognoses in patients with metastatic tumors undergoing chemotherapy and ICI treatment. Further validations, including a prospective cohort study or a functional study, would be particularly valuable in advancing the knowledge on this aspect and developing improved prognostic parameters.

## Graphical abstract

TP53 is the most commonly mutated tumor suppressor gene across all cancer types. We show that four factors determine the response to chemotherapy in patients with metastatic solid tumors harboring the TP53 mutation.TP53 mutations can predict different outcomes following chemotherapy and immunotherapy.

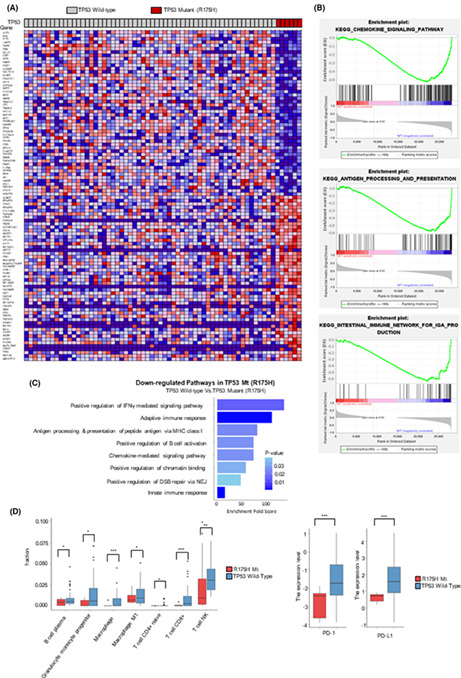

## 1 BACKGROUND

*TP53* is a transcription factor that regulates cellular proliferation, DNA repair, and apoptosis as a tumor suppressor.^1^, ^2^, ^3^ The gene encoding this transcription factor, *TP53*, is the most commonly mutated gene across all cancer types.^4^ Up to 50% of esophageal adenocarcinomas (ADCs), lung squamous cell carcinomas (SqCCs), pancreatic ADCs, and colorectal ADCs harbor *TP53* mutations.^5^, ^6^, ^7^, ^8^, ^9^ *TP53* mutations most frequently occur in the DNA‐binding domain located in amino acid residues 102 and 292.^3^, ^10^, ^11^ Of the several mutational hotspots in *TP53*, eight hotspot mutations constitute 28% of all *TP53* mutations despite missense *TP53* mutations occurring in nearly 190 codons.^10^, ^12^ In colorectal cancer, approximately 37% of all *TP53* mutations were comprised with missense mutations in p53 residues R175, R273, R248, and R282 which were four most commonly mutated.^1^ In this study, we sought new loci which could be influential to survival period. In addition, R175H is known as the most frequent and significant hotspot in *TP53* mutants. The recent studies have suggested that *TP53* can undergo gain‐of‐function (GoF) mutations despite its role as a tumor suppressor gene.^11^, ^13^ Previous research has suggested that ectopic expression of *TP53* with R175H and R273H mutations enhances tumorigenic potential and increases the expression of drug resistance genes.^11^, ^13^, ^14^ R175H mutation was considered structural mutation where the mutation causes misfolding of the protein and leads to a significant conformational alterations within p53's DNA binding domain.^1^ In this study, we tried to explain the reason why R175H worse the response to immunotherapy by analyzing tumor immune microenvironment through the expression of immune cells and PD‐1.

Furthermore, we aimed to analyze the pan‐cancer prevalence and profile of mutations in the *TP53* gene. We also investigated the effect of *TP53* mutations at each amino acid locus and structural domain. We sought to determine the incidence of *TP53* mutations and the association between each *TP53* locus and survival outcome at a pan‐cancer level using a targeted deep sequencing (TDS) panel. Next, we analyzed the impact of *TP53* mutation on treatment response to chemotherapy and immunotherapy.

## 2 METHODS

### 2.1 Study population

**FIGURE 1:**
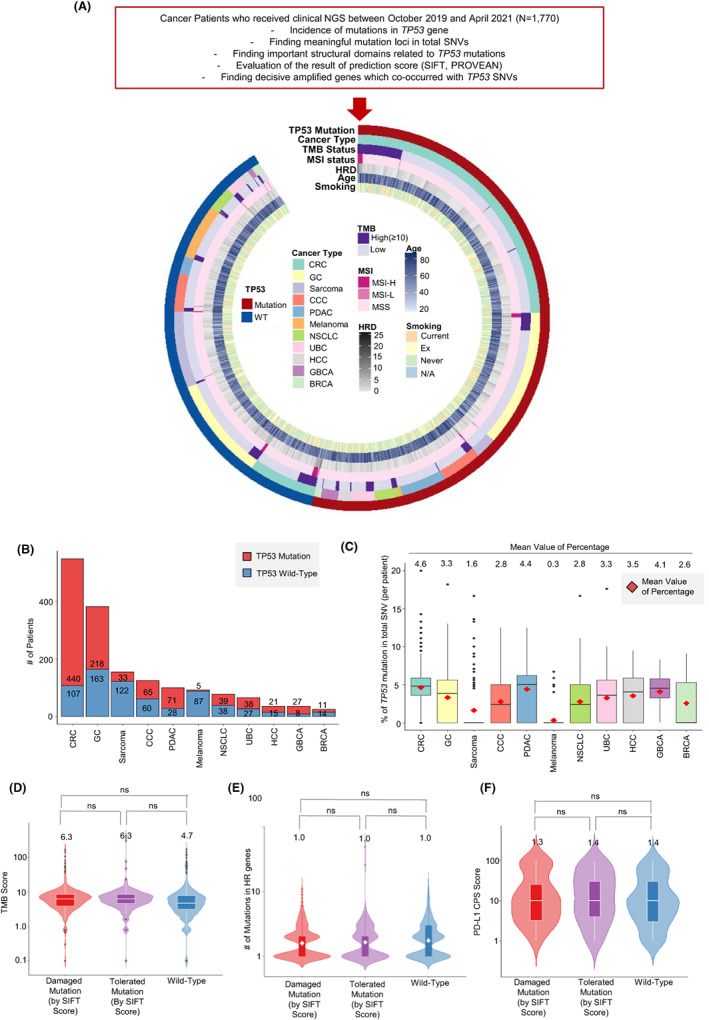
Overview of enrolled cancer patients and incidence of mutations in *TP53*. (A) Flow chart of analysis in this study and comprehensive outline of clinical or pathological features of every person in the cohort. (B) The number of patients with and without *TP53* mutation in each cancer type. (C) The percentage of *TP53* mutations among total SNVs per patient. The red rhombus presents the mean value of percentage. Violin plots show the (D) range of TMB score, (E) number of mutations in HRD genes, and (F) range of PD‐L1 CPS scores between groups (group 1: patients with damaging mutations in *TP53* by SIFT score, group 2: patients with tolerated mutations in *TP53* by SIFT score, group 3: patients without *TP53* mutations). (TMB: tumor mutational burden, CPS: combined positive score, SIFT: Sorting Intolerant from Tolerant)

Patients diagnosed with metastatic carcinoma, including colorectal cancer (CRC), breast cancer (BRCA), gastric cancer (GC), non‐small cell lung cancer (NSCLC), and 20 other cancer types, treated in a palliative setting at Samsung Medical Center between October 2019 and April 2021, were enrolled. Of these patients, those who underwent TDS analysis (TruSight™ Oncology 500 assay [TSO 500]) were finally analyzed (Figure 1A and data supplement). Treatment information regarding immunotherapy, chemotherapy, and survival outcomes, including progression‐free survival (PFS) per line of treatment and overall survival (OS), were collected. For survival analysis, we defined the cohort treated with immune checkpoint inhibitor (ICI) as metastatic cancer patients treated with ICI only or ICI with chemotherapy. ICIs included pembrolizumab, atezolizumab, nivolumab, and duvalumab. Cohort treated with chemotherapy was defined as metastatic cancer patients treated with cytotoxic chemotherapy without ICI. This study was performed in accordance with the principles of the Declaration of Helsinki and the Korean Good Clinical Practice guidelines. The collection of specimens and associated clinical data used in this study was approved by the Institutional Review Board of Samsung Medical Center (IRB File No. 2021–08‐046), and the need for informed consent was waived.

### 2.2 DNA extraction

Metastatic tumor regions were micro‐dissected for most tumor tissues, except for the samples used in genomic DNA extraction. Genomic DNA was isolated from formalin‐fixed paraffin‐embedded (FFPE) tissue fragments and purified using AllPrep DNA/RNA FFPE Kit (Qiagen). DNA concentration was measured using a Qubit dsDNA HS assay kit (Thermo Fisher Scientific), and 40 ng DNA was used as input for library preparation. DNA integrity number, which is a measure of DNA fragment size and consequently DNA quality, was determined using the Genomic DNA ScreenTape assay on an Agilent 2200 TapeStation system (Agilent Technologies).

### 2.3 Library preparation and data analysis

Following the manufacturer's protocol, we prepared DNA library applying a hybrid capture‐based TruSight Oncology 500 DNA/RNA NextSeq Kit. Using unique molecular identifiers (UMIs) in TruSight Oncology 500 (TSO 500), we determined the unique coverage of each position and lowered background noise. In the process of DNA library preparation, we can detect low variant allele frequencies (VAFs) with reducing errors, thereby guaranteeing high specificity.

We analyzed sequencing data for genomic alterations, including SNVs, CNVs, and fusions. SNVs and small indels which of variant allele frequency (VAF) were less than 2% were excluded. Average copy number more than 4 were considered as amplification. Only amplification was analyzed in the TSO 500‐CNV analysis. RNA translocation‐supporting reads of which were more than 4–12 were included in translocation. Annotation of data output from the TSO 500 pipeline (Illumina) were performed using the Ensembl Variant Effect Predictor (VEP) Annotation Engine. The variations were marked according to a 4‐tier system suggested by the American Society of Clinical Oncology/College of American Pathologists. TMB and microsatellite instability (MSI) statuses was determined by TSO 500 pipeline (Illumina).

### 2.4 PROVEAN and SIFT score prediction

To know whether an amino acid substitution or indel cause significant impacts on the biological function of a protein, we used a software tool named PROVEAN.^15^ After files containing information about amino acid substitutions were uploaded, the files showing the prediction results were saved. This analysis also provided SIFT scores with PROVEAN scores.^16^

### 2.5 Sequencing of whole transcriptome

Total RNA concentration and quality were calculated using Quant‐IT RiboGreen (Invitrogen). After samples were run on the TapeStation RNA ScreenTape (Agilent), we used 100 ng of total RNA for sequencing library construction with the TruSeq RNA Access Library Prep Kit (Illumina) following the manufacturer's protocol. After total RNA was fragmented into small pieces, they were copied into first‐strand cDNA using SuperScript II reverse transcriptase (Invitrogen) and random primers, following by second‐strand cDNA synthesis. The purified products were enriched using PCR to make the cDNA library. After normalization and pooling six into a single hybridization/capture reaction, pooled libraries were incubated with biotinylated oligos which were correspond to the coding regions of the genome. Using streptavidin‐conjugated beads, targeted library molecules were collected by hybridized biotinylated oligo probes. The collected libraries were quantified using KAPA Library Quantification kits for Illumina Sequencing platforms following the qPCR Quantification Protocol Guide (KAPA BIOSYSTEMS, #KK4854). Indexed libraries were then subjected to an Illumina HiSeq2500 (Illumina, Inc.), and Macrogen Inc. (South Korea) performed paired‐end sequencing.

### 2.6 Preprocessing of whole‐transcriptome sequencing data

Annotation of RNA sequence were performed with ENSEMBL (version 98) and align to the human reference genome (GRCh38) was conducted using STAR (version 2.6.1; ref. 17). We quantified in units of transcript per million (TPM) by applying RSEM (version 1.3.1; ref. 18) pipeline. TPM values less than 1 were considered as zero.

### 2.7 Profiling Gene Set Activity in Whole‐Transcriptome Sequencing

To explore the activity of signaling pathways, we used gene set variation analysis (GSVA^18^) and GSEA program in default mode. Enrichment analysis was performed using DAVID (Database for Annotation, Visualization, and Integrated Discovery) (https://david.ncifcrf.gov/). The calculation of composition of immune cells was performed by xCell using immunedeconv R package.

### 2.8 Statistical analysis

Descriptive statistics are reported as proportions and medians. Data are presented as number (%) for categorical variables. Correlations between clinical characteristics and TP53 status were analyzed by a *t*‐test, Fisher's exact test, chi‐square test, or two‐way analysis of variance, as appropriate. OS was defined as the time from the first treatment to the date of death. PFS was defined as the time from treatment initiation to the date of disease progression or all‐cause mortality. Kaplan–Meier estimates were used in the analysis of all time‐to‐event variables, and the 95% confidence interval (CI) was computed for the median time to an event. All statistical analyses were performed using R for Windows (version 4.1.2, https://cran.r‐project.org/bin/windows/base/). RStudio desktop was used to draw all graphics (RStudio Team, Boston, MA, USA; https://www.rstudio.com/products/rstudio/download/). The following R packages were used for graphics: ggplot2 (3.3.5), Ggally (2.1.2), corrplot (0.92), Polychrome (1.4.0), biomaRt (2.48.3), survival (3.2–13), survminer (0.4.9), ggpubr (0.4.0), pylr (1.8.6), dplyr (1.0.7), RcolorBrewer (1.1–2), and scatterpie (0.1.7).

## 3 RESULTS

### 3.1 *TP53* mutation profiles

TSO 500 data from 1770 patients were included in the genomic data analysis (Figure 1A). Of 1770 patients, 1012 (57.2%) harbored genetic alterations in *TP53*. *TP53* mutation, cancer type, TMB, MSI, HRD, age at cancer diagnosis, and smoking status are summarized in Figure 1A. In patients having both wild‐type and mutational *TP53*, we evaluated 547 (30.9%) cases of CRC, 381 (21.5%) cases of GC, 155 (8.8%) cases of sarcoma, and 125 (7.1%) cases of cholangiocarcinoma (CCC), and 99 (5.6%) cases of pancreatic ductal adenocarcinoma (PDAC) (Figure S1).

*TP53* mutation was most frequently observed in CRC (440/547, 80.4%), followed by gall bladder cancer (GBCA) (27/35, 77.1%), PDAC (71/99, 71.7%), urothelial cancer of the bladder (UCB) (38/65, 58.5%), hepatocellular carcinoma (HCC) (21/36, 58.3%), and GC (218/381, 57.2%), whereas only 5.4% (5/92) of melanomas and 21.3% (33/155) of sarcomas harbored *TP53* mutations (Figure 1B). To investigate how much *TP53* SNVs occupied in total SNV in each cancer type, we calculated the percentage of *TP53* mutation in total SNVs per sample (Figure 1C). The number of *TP53* mutations per total SNVs showed CRC, PDAC, and GBCA to have *TP53* mutation frequencies of 4.6%, 4.4%, and 4.1% of all SNVs, respectively (Figure 1C). Additional analysis presenting the *TP53* mutation loci in each cancer type was displayed in Figure S1. In individual loci of *TP53* mutation, the most frequently mutated locus was R175H (*n* = 84, 7.5%), followed by R273H (*n* = 46, 4.1%), R248Q (*n* = 43, 3.8%), R282W (*n* = 37, 3.3%), and R273C (*n* = 33, 2.9%) (Figure S1).

Before detailed analysis, we also categorized *TP53* mutations into damaging and tolerated mutations according to SIFT predictions,^15^, ^16^ and further analysis surrounding the relationship between *TP53* mutation and TMB, HRD, and PD‐L1 (combined positive score: CPS) status was performed. The median values of TMB scores were 6.3, 6.3, and 4.7 in damaging mutation, tolerated mutation, and wild‐type *TP53* groups, respectively (Figure 1D). The median number of mutations in HRD genes was 1.0 in all groups (Figure 1E), whereas the median CPS scores were 1.3, 1.4, and 1.4, respectively, in each group (Figure 1F).

### 3.2 Prognostic value of each *TP53* mutation locus

**FIGURE 2:**
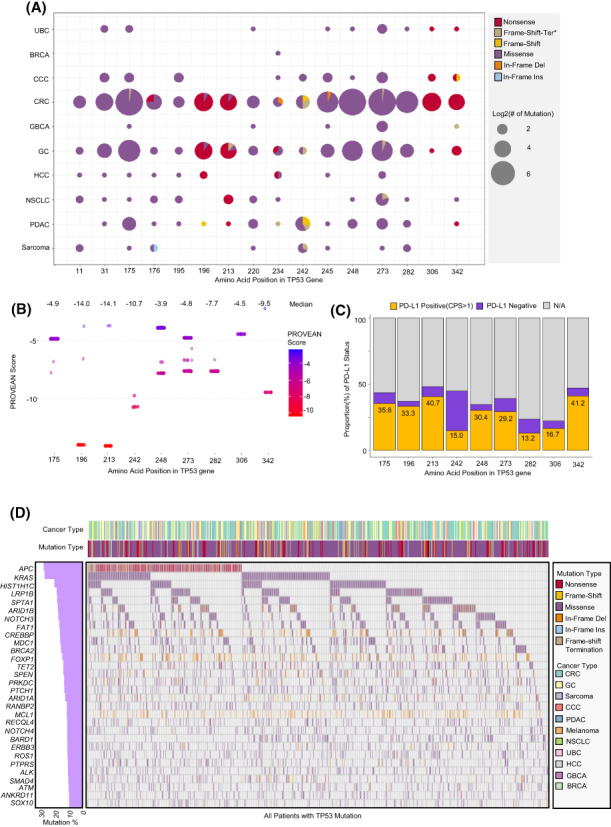
Prognostic value of each *TP53* mutation locus. (A) Bubble chart showing the number of mutations (proportional to circle size) and proportion of mutational type (colored part of the circle) in the most frequent mutational loci (top 16) in various cancer types. (B) Dot plots representing the PROVEAN score by each mutational locus. The median values of scores are indicated at the top of the graph. (C) The proportion of PD‐L1 status in the patients with *TP53* mutations at each locus (positive: CPS ≥1, negative <1). The percentage of PD‐L1‐positive patients is indicated in the yellow box in the chart. (D) Oncoprint of top SNV genes in patients harboring *TP53* mutations. (upper panel: cancer type and the type of mutation in the *TP53* gene; lower panel: oncoprint showing mutational types of each gene in each individual; left panel: percentage of SNVs of each gene in the patients with *TP53* mutations).

Details of mutation loci among cancer types are described in Figure 2A. Most *TP53* mutations were missense mutations, whereas nonsense mutations were frequently observed in R196 (*n* = 24/27, 88.9%), R213 (*n* = 24/27, 88.9%), R306 (*n* = 18/18, 100%), and R342 (*n* = 15/18, 83.3%) loci (Figure 2A). To identifying which loci mutation was influential to survival period, we performed survival analysis with all loci presented in Figure 2A. As a result, the patients having mutation in R213, R342, C242, and R282 had shorter survival period than those having mutation in other loci. (Figure S2). In addition, we assessed the prognostic value of the *TP53* R175H mutation, which is a well‐established marker of poor prognosis. We found that the OS and PFS of patients with R175H were shorter than those of other SNVs following both chemotherapy and ICI treatment (Figure S2).

Therefore, we evaluated the biological functional damage of mutation in each loci by calculating PROVEAN score. The PROVEAN score of each locus showed that R196 and R231 loci had lower scores (median PROVEAN score: −14.0 and 14.1, respectively) than the other loci (median: −5.8) (*p* < 0.001) (Figure 2B). In addition, C242 and R342 loci had relatively lower scores (median: −10.7 and −9.5, respectively) than other loci (*p* < 0.001) (Figure 2B). We further analyzed the relationship between PD‐L1 status and *TP53* mutation loci in 187 cancers treated with immune checkpoint inhibitors (ICIs) (Figure 2C). In this analysis, PD‐L1 positivity defined as CPS ≥1 was associated with *TP53* R342 (41.2%) and R213 (40.7%) mutations, whereas PD‐L1 positivity was inversely associated with C242 (15.0%) and R282 (13.2%) mutations (*p* < 0.001). There was not clear correlation between PD‐L1 and loci, although each loci has various PD‐L1 positivity depending on mutation site. These results suggested the loci of mutation could affect PD‐L1 status. In the patients harboring *TP53* mutations, the most frequently co‐occurring mutation was found in *APC*, followed by *KRAS*, *HIST1H1C*, *LRP1B*, and *SPTA1* (Figure 2D).

### 3.3 The difference of gene expression profile as a factor contributing to survival in R175H mutation

**FIGURE 3:**
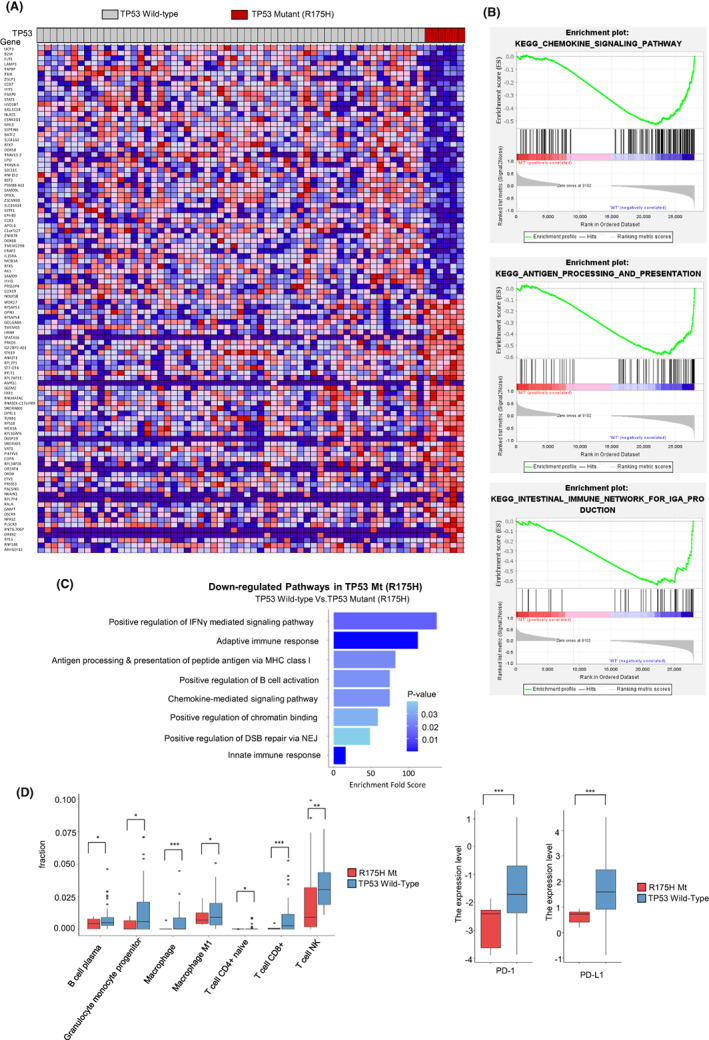
Analysis of transcriptomic profile related to immune environment in tumor sample. (A) The heatmap of gene (*n* = 100) expression of 64 CRC patients. R175H patients (*n* = 6) were ordered at the right of heatmap. (B) GSEA results presenting downregulated pathways in R175H patients. (C) Fold Enrichment score from 50 genes which were downed in R175H patients using DAVID tools. (D) The comparison of the number of immune cells by xCell program. (E) The expression level of PD‐1 and PD‐L1 in R175H mutant tumors and WT group samples.

It is known that there are some hotspots in *TP53* mutations. Among them, although R175H mutations is most frequent mutation, both the correlation between R175H mutation and the result of immunotherapy and the changes of tumor immune microenvironment by R175H mutation have not yet been sufficiently reported. In an effort to elucidate potential transcriptional signature or molecular determinants that are associated with response to ICI therapy, we compared gene expression profiles for tumors having R175H mutation in *TP53* with *TP53* wild‐type tumors. In total 64 CRC tumors, six had R175H in *TP53* gene. The genes (*n* = 100) with the greatest difference in their expression between two groups were presented as the heat map (Figure 3A).In fold enrichment analysis by GSEA, chemokine signaling pathway was reduced in tumors having R175H mutation compared to wild‐type tumors (normalized enrichment score: −1.83, false discovery rate[FDR] q‐value: 0.004) (Figure 3B upper panel). In addition, antigen‐presenting pathway (normalized enrichment score: −1.83, FDR q‐value: 0.004) and immune network for IgA production pathway (normalized enrichment score:‐1.87, FDR q‐value: 0.003) were downregulated in R175H mutation patients (Figure 3B middle and lower panel).

With 50 genes whose expression was the most lowered in R175H mutated tumors, we performed the analysis of fold enrichment pathways with DAVID bioinformatics tools (Figure 3C). As a result, following pathways were downregulated in R175H‐mutated tumors: adaptive immune response (*p* < 0.001), antigen processing and presentation of peptide antigen via MHC class I (*p* = 0.024), positive regulation of B‐cell activation (*p* = 0.024), chemokine‐mediated signaling pathway (*p* = 0.025), innate immune response (*p* < 0.001) (Figure 3C). We also analyzed immune cell abundance base on expression of gene related to immunity using xCell. Especially, Plasma B cells (*p* = 0.032), granulocyte monocyte progenitor cells (*p* = 012), macrophages (*p* < 0.001), M1 macrophages (*p* = 0.017), naïve CD4(+) T cells (*p* = 0.042), CD8(+) T cells (*p* = 0.008), NK T cells (*p* = 0.005) were significantly lowered in R175H patients (Figure 3D). According to previous article, PD‐1 expression balance between CD8 + T cells and Treg cells predicts the clinical efficacy of PD‐1 blockade therapies. Therefore, we checked PD‐1 and PD‐L1 expression by RNA‐seq, showing the expression of two genes were significantly lowered in R175H mutant samples (*p* = 0.002 and *p* < 0.001) (Figure 3E). The reduction in immune cells and the expression of PD‐1 could explain some part of reason why R175H mutation patients had shorter survival period to immunotherapy.

### 3.4 Correlation between survival and functional domains and prediction of *TP53* mutations

**FIGURE 4:**
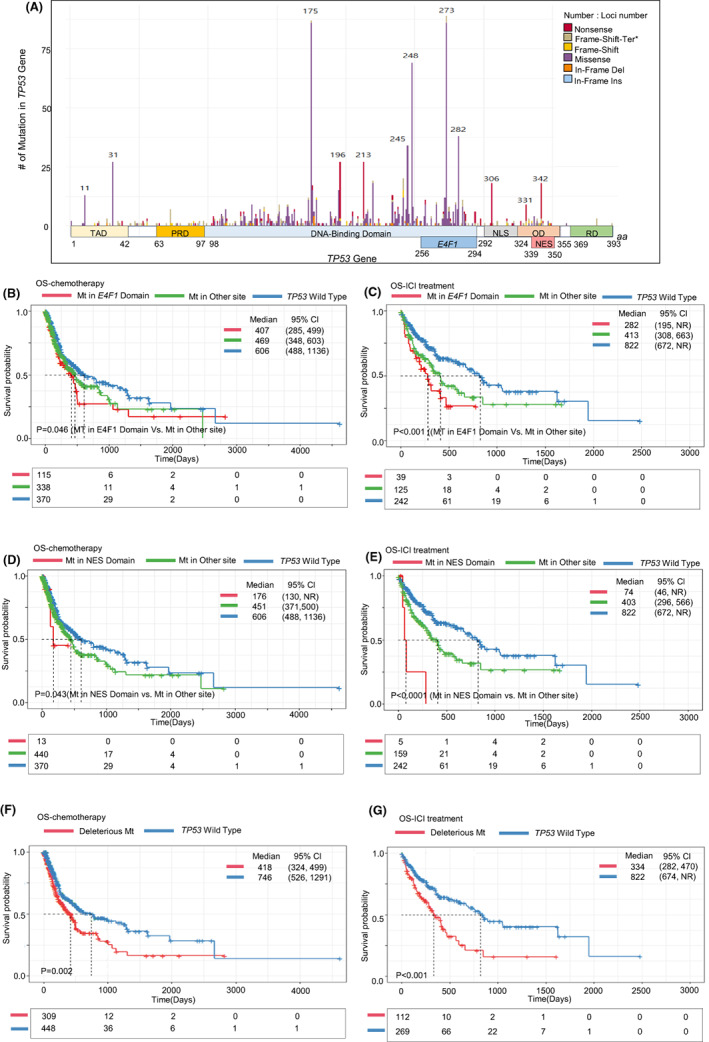
Analysis of *TP53* mutations by functional domain and the effects of *TP53* mutations on overall survival (OS) by the degree of damage in protein function and specific mutational loci. (A) Infographic chart representing the structural functional domain in the *TP53* gene and the number of mutations at every amino acid position. Bar color shows the type of mutation in terms of amino acid substitution. Bar height indicates the number of mutations. Kaplan–Meier‐estimated OS curve in the patients treated with chemotherapy (B, D) and ICI (C, E). The bottom box represents the number of patients at each time (days). (red line: group with mutations in the E4F1‐binding domain (B, C) or NES domain (D, E); green line: group with mutations in other sites; blue line: group with normal *TP53* gene). Kaplan–Meier‐estimated OS curve in the patients treated with chemotherapy (F) or ICI (G). (red line: group with deleterious mutations in *TP53* by PROVEAN score (< −2.5); blue line: group with normal *TP53* gene).

*TP53* mutations were categorized according to the functional domain affected by the mutation (Figure 4A). Most *TP53* mutations were in the DNA‐binding domain (DBD) (*n* = 681, 65.0%), followed by the E4F1‐binding domain (*n* = 251, 24.0%) (Figure S3). Across the observed nonsense mutations, the DBD (*n* = 98, 14.4%) and the nuclear export signal (NES) domain (*n* = 17, 54.8%) were most commonly affected (Figure S3). Additional analysis showed that there were no differences in the type of mutation or functional domains among cancer types (Figure S3). According to survival analysis, *TP53* mutations in the E4F1 domain were associated with short OS following both chemotherapy and ICI treatment (median OS following chemotherapy [days]: 407, 469 for others, and 606 for wild type, *p* = 0.046; median OS following ICI [days]: 282, 413, and 822; *p* < 0.001) (Figure 4B, C). In addition, *TP53* mutations in the NES domain were related to poor OS outcomes compared to the other domains (median OS following chemotherapy [days]: 176, 451 for other domains, and 606 for the wild type, *p* = 0.043; median OS for ICI [days]: 74, 403, and 822; *p* < 0.001) (Figure 4D, E).

We categorized *TP53* mutations by SIFT and PROVEAN predictions (Figure S3). According to SIFT predictions, BRCA and GBCA most frequently harbored damaging *TP53* mutations (BRCA, *n* = 8, 100%; GBCA, *n* = 19, 100%), whereas GBCA most frequently harbored deleterious mutations according to PROVEAN scores (*n* = 18, 94.7%) (Figure S3). Regarding the distributions of SIFT and PROVEAN scores, the SIFT scores of *TP53* mutations were concentrated under 0.05 (*n* = 690, 75.1%), whereas PROVEAN scores were in the range of −20 to 0 points (*n* = 910, 99.0%) (Figure S3). The relationship between SIFT and PROVEAN scores is described in Figure S3. In addition, we calculated the impact of *TP53* mutation PROVEAN scores on the response to chemotherapy (Figure S3). Despite statistical insignificance (median score of PROVEAN in complete response [CR], partial response [PR], stable disease [SD], and progressive disease [PD]: −5.3, −5.9, −5.7, and −5.8, respectively; *p* = 0.582), tumors with extremely low *TP53* mutation PROVEAN scores were more frequently observed in SD and PD than in CR and PR (Figure S3).

Further survival analysis was performed on PROVEAN predictions of *TP53* mutation. Tumors with deleterious *TP53* mutations were associated with shorter OS than those with neutral or no *TP53* mutations in both patients who underwent chemotherapy (median OS [days] for 770 patients: 418 vs. 746; *p* = 0.002) (Figure 4F) as well as patients who underwent immunotherapy (median OS [days] for 187 patients: 334 vs. 822; *p* < 0.001) (Figure 4G).

### 3.5 Copy number variants related to *TP53* mutation

**FIGURE 5:**
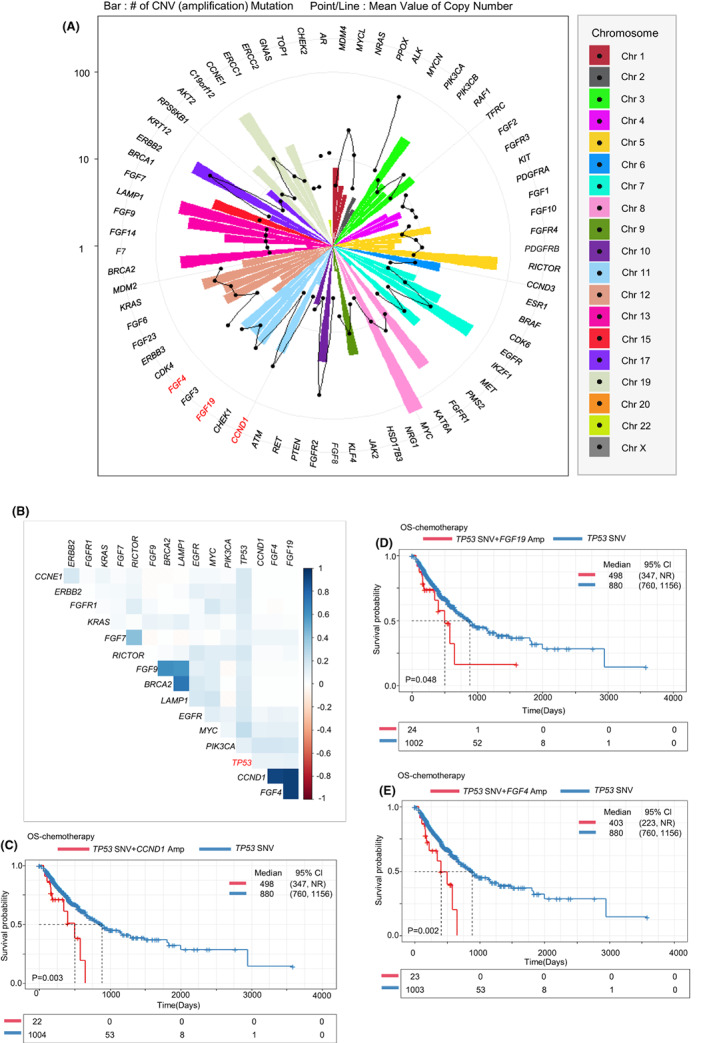
Copy number variants in patients with *TP53* mutations and their effect on overall survival. (A) Circled bar graph representing the genes in which amplification occurred in patients with *TP53* mutations. Bar height indicates the number of CNVs in each gene. The black point in the middle of the bar shows the mean value of copy number in each gene. The genes are ordered and colored by chromosome number. (B) Correlation between the occurrence of *TP53* mutation and the occurrence of each CNV. CNVs represented in the graph indicate the most frequent CNVs among all CNVs (frequency ≥20). Kaplan–Meier‐estimated OS curve in patients treated with chemotherapy who have copy number amplifications in *CCND1* (C), *FGF19* (D), and *FGF4* (E) genes (red line: group with CNVs in specific genes and SNVs in the *TP53* gene; blue line: group with SNVs only in the *TP53* gene).

Copy number variants (CNVs) associated with *TP53* mutations were evaluated. Across copy number variations, only amplifications (copy number ≥4) were reported according to the our hospital reporting system (deletions were not reported). In patients with *TP53* mutation, 69 genes were amplified in total. Among them, *MYC* (*n* = 132), *RICTOR* (*n* = 73), *EGFR* (*n* = 68), *CCNE1* (*n* = 64), *LAMP1* (*n* = 61), and *BRCA2* (*n* = 54) were the most frequently amplified genes in cancers harboring *TP53* mutations (Figure 5A). Among these CNV events, *CCND1*, *FGF4*, and *FGF19* in chromosome 11 were strongly related (Figure 5B). According to survival analysis, tumors with *TP53* mutation and co‐existing copy number amplification of the three aforementioned genes conferred poor survival outcomes compared to those with only *TP53* mutation following chemotherapy (*p* < 0.05) (Figure 5C–E). However, there was no such significant difference pertinent to ICI treatment (data not shown).

### 3.6 Features of good responses to chemotherapy according to machine learning

Finally, we tried to predict the response to chemotherapy using the neuralnet R package. A neural network is a computational system that creates predictions based on existing data. A neural network consists of input layer, hidden layer, and output layer. The model was designed to have five hidden layers with a threshold of 0.01 (Figure S4). We tested the number of hidden layer from 1 to 10, and we chose 5 hidden layers because prediction rate was highest. We divided our data to 80% for training set and 20% for test set. We input (1) the CNV data of *CCND1*, *FGF4*, and *FGF19*; (2) *TP53* hotspot mutations (C242, R282, R342, and R175); (3) deleterious mutations predicted by PROVEAN; and (4) *TP53* NES or E4F1‐binding domain mutations and predicted the treatment response (CR/PR or SD/PD). Among the four factors, the *TP53* mutation domain was the most weighted (0.35), followed by PROVEAN score (0.28), *TP53* loci (0.24), and CNV (0.14) (Figure S4). The accuracy of this model was 0.84 (95% CI: 0.8035, 0.8637); its sensitivity was 1.00, and its specificity was 0.59 (Figure S4), showing this model's sensitivity is very high. According to this model, the most important factor determining the response to chemotherapy was the domain was involved in *TP53* mutation (Figure S4). Next, the response is determined by whether the mutation is predicted as deleterious or not as determined by the PROVEAN score. The third factor was the presence of the mutation locus in a hotspot. The final factor was co‐occurrence with the amplification of *FGF19*, *FGF4*, or *CCND1* (Figure S4).

## 4 DISCUSSION

*TP53* is a well‐known tumor suppressor gene, but its mechanisms of action are complex. *TP53* is involved in genomic instability, angiogenesis, replicative cell immortality, tumor invasion, and tumor‐promoting inflammation.^19^, ^20^, ^21^, ^22^, ^23^ In this study, we investigated the prevalence and profile of *TP53* mutations in patients with different types of cancers who underwent clinical NGS analysis. In addition, we analyzed genomic mutational factors affecting the survival of these patients. All genomic mutations were investigated after dividing them according to mutational locus (amino acid position), structural domains, and degree of functional protein damage. Lastly, we developed a machine learning model that could predict the response to chemotherapy/immunotherapy and calculate the importance of each factor. According to our model, domain harboring mutation was the most important factor which could determine the response of chemotherapy/immunotherapy, following by PROVEAN score, loci where mutation occurred and co‐occurring CNVs.

First, in terms of domain, structural domain analysis showed that mutations occurring in the NES or E4F1‐binding domain were critical for survival after both chemotherapy and immunotherapy. The NES domain exports protein and the attached RNA into the cytoplasm through the nuclear pore.^24^ Many studies have demonstrated the importance of the NES domain in proteins related to cancer.^25^, ^26^, ^27^ For example, EGFR NES mutants with increased nuclear accumulation promote the aggressiveness of cancer cells, including TKI resistance.^28^ Moreover, single‐allele mutations in the EGFR NES domain are capable of driving lymphomagenesis, suggesting that the EGFR NES mutant possesses strong oncogenic activity.^28^ Similar to this mechanism, TP53 proteins bearing mutations in the NES domain cannot export proteins to the cytoplasm, which accumulate in the nucleus and block the function of TP53. E4F1 is a key posttranslational regulator of TP53, which modulates its effector functions involved in alternative cell fates: growth arrest or apoptosis. E4F1 mediates mutually exclusive posttranslational modifications of TP53.^29^ E4F1‐dependent Ub–TP53 conjugates are associated with chromatin, and their stimulation coincides with the induction of a TP53‐dependent transcriptional program specifically involved in cell cycle arrest and not apoptosis.^29^ According to this study, mutations in the E4F1‐binding domain of TP53 interfere with posttranslational modifications of TP53, suggesting loss of TP53 function.

In terms of loci, Specifically, missense mutations in the four most commonly mutated p53 residues (R175, R248, R273, and R282) comprise most of all TP53 mutations. Among these hotspot mutations, p53‐R175H has the highest occurrence. Although losing the transactivating function of the wild‐type p53 and prone to aggregation, p53‐R175H gains oncogenic functions by interacting with many proteins. We found another important loci (R213, R342, C242, and R282) which can cause short survival period. Our study also suggested that the R175H mutation was associated with shorter PFS and OS than other mutations and wild‐type *TP53* following both chemotherapy and ICI treatment. Recent studies have suggested that *TP53* can undergo GoF mutations despite its role as a tumor suppressor gene.^11^, ^13^ Ectopic expression of *TP53* with the R175H mutation enhances tumorigenic potential and increases the expression of drug resistance genes.^11^, ^13^, ^14^ The R175H mutation regulates doxorubicin‐induced apoptosis.^30^, ^31^ *TP53* R175H mutations in lung cancer result in drug resistance against etoposide and cisplatin.^32^ Functionally, tumors harboring *TP53* GoF mutations have been found to progress more rapidly and confer poorer survival outcomes than tumors with deleterious *TP53* mutations.^33^, ^34^ In this study, we tried to look for the reason why R175H mutation made the response to chemotherapy/immunotherapy worse by analyzing tumor microenvironment using RNA‐seq. In RNA sequencing data, we found that the expression of TP53 was elevated in *TP53* R175H compared to wild‐type and GoF of *TP53* could exert on tumor environment. By analysis GO term related differentially expressed gene between normal and R175H mutation patients, both innate and adaptive immune response pathways including cytokine‐mediated signaling pathway were lowered in R175H‐mutant tumors. Furthermore, some immune cells proportions were significantly lowered in R175H patients. In addition, PD‐1 and PD‐L1 expression were also significantly lower in R175H mutant tumors compared to others. According to previous article, PD‐1 expression balance between CD8 + T cells and Treg cells predicts the clinical efficacy of PD‐1 blockade therapies. The reduction of immune cells and the expression of PD‐1 could explain some part of reason why the response to immunotherapy in patients harboring *TP53* R175H mutation was worse than patients with wild‐type *TP53*. Moreover, a recent study targeting the R175H locus of *TP53* revealed a cancer treatment strategy including therapeutic antibodies targeting neoantigens derived from a common *TP53* mutation.^35^ We suggest that other loci of *TP53* mutations might also be effective targets for T‐cell therapy in the near future.

We also focused on copy number amplification in tumors harboring *TP53* mutations. Interestingly, *FGF4*, *FGF19*, and *CCND1* copy number amplifications were associated with each other. These three copy number amplifications with *TP53* mutations conferred poor survival outcomes following chemotherapy. Interestingly, chromosome 11 allelotypes and *TP53* have been reported to collaborate to induce chemical carcinogenesis.^36^, ^37^ Further functional research will confirm the interaction between *TP53* and gene amplification in chromosome 11.

Our study had a limitation. In our database, *TP53* R175H mutation had worse prognosis in patients treated with immune checkpoint inhibitor. However, we could not validate our result. We had tried to validate our finding for a predictive role of R175H mutation but public database including the cancer genome atlas did not have treatment information of immune checkpoint inhibitor. Moreover, we had tried to search the references for specific *TP53* genetic alterations and immunotherapy, but there were few articles to analyze the relationship between *TP53* genetic alteration and immunotherapy. Further functional study would be warranted to support our data.

Overall, we found each *TP53* mutation to indicate different prognoses in patients with metastatic tumors undergoing chemotherapy and ICI treatment. This information would be of great value for the determination of patient prognosis in the metastatic setting. The model showed high sensitivity and specificity. We expect to improve this model by collecting and applying more data in the future. Further validations, including a prospective cohort study or a functional study, would be particularly valuable in advancing the knowledge on this aspect and developing improved prognostic parameters.

## AUTHOR CONTRIBUTIONS

**Ji‐Yeon Kim:** Conceptualization (lead); data curation (equal); formal analysis (lead); funding acquisition (equal); writing – original draft (lead); writing – review and editing (equal). **Jaeyun Jung:** Data curation (lead); formal analysis (lead); methodology (lead); resources (equal); software (lead); writing – original draft (equal); writing – review and editing (equal). **Kyoung‐Mee Kim:** Methodology (equal); resources (equal); supervision (equal). **Jeeyun Lee:** Project administration (lead); supervision (lead); writing – original draft (equal); writing – review and editing (lead). **Young‐Hyuck Im:** Investigation (equal); project administration (equal); supervision (lead); writing – original draft (equal); writing – review and editing (equal).

## FUNDING INFORMATION

This work was supported by a grant from the Korea Health Technology R&D Project through the Korea Health Industry Development Institute (KHIDI) funded by the Ministry of Health & Welfare, Republic of Korea (grant number: HR20C0025), a grant from Samsung Medical Center (SMO1220551), and grants from the National Research Foundation of Korea (NRF‐2017R1D1A1B03028446, NRF‐2020R1F1A1072616).

## CONFLICT OF INTEREST STATEMENT

There are no conflicts of interest to declare.

## ETHICS STATEMENT

Approval of the research protocol by an Institutional Reviewer Board: The collection of specimens and associated clinical data used in this study was approved by the Institutional Review Board of Samsung Medical Center (IRB File No. 2021–08‐046).

## INFORMED CONSENT

The need for informed consent was waived.

## REGISTRY AND THE REGISTRATION NO. OF THE STUDY/TRIAL

N/A.

## ANIMAL STUDIES

N/A.

## DATA AVAILABILTY STATEMENT

The dataset used for the current study is available from the corresponding author upon reasonable request.

## Supporting information


Figure S1.

Figure S2.

Figure S3.

Figure S4.

## References

[1] Hassin O , Nataraj NB , Shreberk‐Shaked M , et al. Different Hotspot p53 Mutants Exert Distinct Phenotypes and Predict Outcome of Colorectal Cancer Patients. 2022;13:1‐15. 10.1038/s41467-022-30481-7 PMC9120190 35589715

[2] Finlay CA , Hinds PW , Levine AJ . The p53 proto‐oncogene can act as a suppressor of transformation. Cell. 1989;57:1083‐1093. 2525423 10.1016/0092-8674(89)90045-7

[3] Vousden KH . Activation of the p53 tumor suppressor protein. Biochim Biophys Acta. 2002;1602:47‐59. 11960694 10.1016/s0304-419x(02)00035-5

[4] Muller PA , Vousden KH . p53 mutations in cancer. Nat Cell Biol. 2013;15:2‐8. 23263379 10.1038/ncb2641

[5] Sanchez‐Vega F , Mina M , Armenia J , et al. Oncogenic signaling pathways in the cancer genome Atlas. Cell. 2018;173:321‐337. e310. 29625050 10.1016/j.cell.2018.03.035 PMC6070353

[6] Dulak AM , Stojanov P , Peng S , et al. Exome and whole‐genome sequencing of esophageal adenocarcinoma identifies recurrent driver events and mutational complexity. Nat Genet. 2013;45:478‐486. 23525077 10.1038/ng.2591 PMC3678719

[7] Cancer Genome Atlas Research N . Comprehensive genomic characterization of squamous cell lung cancers. Nature. 2012;489:519‐525. 22960745 10.1038/nature11404 PMC3466113

[8] Bailey P , Chang DK , Nones K , et al. Genomic analyses identify molecular subtypes of pancreatic cancer. Nature. 2016;531:47‐52. 26909576 10.1038/nature16965

[9] Cancer Genome Atlas N . Comprehensive molecular characterization of human colon and rectal cancer. Nature. 2012;487:330‐337. 22810696 10.1038/nature11252 PMC3401966

[10] Baugh EH , Ke H , Levine AJ , Bonneau RA , Chan CS . Why are there hotspot mutations in the TP53 gene in human cancers? Cell Death Differ. 2018;25:154‐160. 29099487 10.1038/cdd.2017.180 PMC5729536

[11] Muller PA , Vousden KH . Mutant p53 in cancer: new functions and therapeutic opportunities. Cancer Cell. 2014;25:304‐317. 24651012 10.1016/j.ccr.2014.01.021 PMC3970583

[12] Brosh R , Rotter V . When mutants gain new powers: news from the mutant p53 field. Nat Rev Cancer. 2009;9:701‐713. 19693097 10.1038/nrc2693

[13] Zhang C , Liu J , Xu D , Zhang T , Hu W , Feng Z . Gain‐of‐function mutant p53 in cancer progression and therapy. J Mol Cell Biol. 2020;12:674‐687. 32722796 10.1093/jmcb/mjaa040 PMC7749743

[14] Dittmer D , Pati S , Zambetti G , et al. Gain of function mutations in p53. Nat Genet. 1993;4:42‐46. 8099841 10.1038/ng0593-42

[15] Choi Y , Chan AP . PROVEAN web server: a tool to predict the functional effect of amino acid substitutions and indels. Bioinformatics. 2015;31:2745‐2747. 25851949 10.1093/bioinformatics/btv195 PMC4528627

[16] Kumar P , Henikoff S , Ng PC . Predicting the effects of coding non‐synonymous variants on protein function using the SIFT algorithm. Nat Protoc. 2009;4:1073‐1081. 19561590 10.1038/nprot.2009.86

[17] Dobin A , Davis CA , Schlesinger F , et al. STAR: Ultrafast Universal RNA‐Seq Aligner. 2013;29:15‐21. 10.1093/bioinformatics/bts635 PMC3530905 23104886

[18] Li BJBB , Dewey CN . RSEM: Accurate Transcript Quantification from RNA‐Seq Data with or without a Reference Genome. 2011;12:323. 10.1186/1471-2105-12-323 PMC3163565 21816040

[19] Lee S , Elenbaas B , Levine A , Griffith J . p53 and its 14 kDa C‐terminal domain recognize primary DNA damage in the form of insertion/deletion mismatches. Cell. 1995;81:1013‐1020. 7600570 10.1016/s0092-8674(05)80006-6

[20] Hendrix MJ . De‐mystifying the mechanism(s) of maspin. Nat Med. 2000;6:374‐376. 10742136 10.1038/74624

[21] Jenkins JR , Rudge K , Currie GA . Cellular immortalization by a cDNA clone encoding the transformation‐associated phosphoprotein p53. Nature. 1984;312:651‐654. 6095117 10.1038/312651a0

[22] Wang SP , Wang WL , Chang YL , et al. p53 controls cancer cell invasion by inducing the MDM2‐mediated degradation of slug. Nat Cell Biol. 2009;11:694‐704. 19448627 10.1038/ncb1875

[23] Gudkov AV , Gurova KV , Komarova EA . Inflammation and p53: a tale of two stresses. Genes Cancer. 2011;2:503‐516. 21779518 10.1177/1947601911409747 PMC3135644

[24] Stommel JM , Marchenko ND , Jimenez GS , Moll UM , Hope TJ , Wahl GMJTEj . A Leucine‐Rich Nuclear Export Signal in the p53 Tetramerization Domain: Regulation of Subcellular Localization and p53 Activity by NES Masking. 1999;18:1660‐1672. 10.1093/emboj/18.6.1660 PMC1171253 10075936

[25] Derheimer FA , Chang C‐W , Ljungman MJEJoC . Transcription Inhibition: A Potential Strategy for Cancer Therapeutics. 2005;41:2569‐2576. 10.1016/j.ejca.2005.08.012 16213135

[26] Gravina GL , Senapedis W , McCauley D , et al. Nucleo‐Cytoplasmic Transport as a Therapeutic Target of Cancer. 2014;7:1‐9. 10.1186/s13045-014-0085-1 PMC4272779 25476752

[27] Kau TR , Way JC . Silver PAJNRCNuclear Transport and Cancer: From Mechanism to Intervention. 2004;4:106‐117. 10.1038/nrc1274 14732865

[28] Nie L , Hou J , Chu Y‐Y , et al. Compartmentalized Functions of Epidermal Growth Factor Receptor (EGFR) in Tumorigenesis and Malignant Phenotypes. 2021;81:120.

[29] Le Cam L , Linares LK , Paul C , et al. E4F1 Is an Atypical Ubiquitin Ligase that Modulates p53 Effector Functions Independently of Degradation. 2006;127:775‐788. 10.1016/j.cell.2006.09.031 17110336

[30] Wong RP , Tsang WP , Chau PY , Co NN , Tsang TY , Kwok TT . p53‐R273H gains new function in induction of drug resistance through down‐regulation of procaspase‐3. Mol Cancer Ther. 2007;6:1054‐1061. 17363498 10.1158/1535-7163.MCT-06-0336

[31] Tsang WP , Ho FY , Fung KP , Kong SK , Kwok TT . p53‐R175H mutant gains new function in regulation of doxorubicin‐induced apoptosis. Int J Cancer. 2005;114:331‐336. 15578696 10.1002/ijc.20818

[32] Blandino G , Levine AJ , Oren M . Mutant p53 gain of function: differential effects of different p53 mutants on resistance of cultured cells to chemotherapy. Oncogene. 1999;18:477‐485. 9927204 10.1038/sj.onc.1202314

[33] Hanel W , Marchenko N , Xu S , Yu SX , Weng W , Moll U . Two hot spot mutant p53 mouse models display differential gain of function in tumorigenesis. Cell Death Differ. 2013;20:898‐909. 23538418 10.1038/cdd.2013.17 PMC3679454

[34] Schulz‐Heddergott R , Stark N , Edmunds SJ , et al. Therapeutic ablation of gain‐of‐function mutant p53 in colorectal cancer inhibits Stat3‐mediated tumor growth and invasion. Cancer Cell. 2018;34:298‐314. e297. 30107178 10.1016/j.ccell.2018.07.004 PMC6582949

[35] Hsiue EH , Wright KM , Douglass J , et al. Targeting a neoantigen derived from a common TP53 mutation. Science. 2021;371:1‐14. 10.1126/science.abc8697 PMC8208645 33649166

[36] Hulla JE , French JE , Dunnick JK . Chromosome 11 allelotypes reflect a mechanism of chemical carcinogenesis in heterozygous p53‐deficient mice. Carcinogenesis. 2001;22:89‐98. 11159746 10.1093/carcin/22.1.89

[37] Anderson MJ , Casey G , Fasching CL , Stanbridge EJ . Evidence that wild‐type TP53, and not genes on either chromosome 1 or 11, controls the tumorigenic phenotype of the human fibrosarcoma HT1080. Genes Chromosomes Cancer. 1994;9:266‐281. 7519049 10.1002/gcc.2870090407

