# Supplementary figures and images for: TP53 mutations predict poor response to immunotherapy in patients with metastatic solid tumors
Source: Cancer Med. 2023 Apr 20;12(11):12438–51. doi: 10.1002/cam4.5953 (PMC10278489; doi:10.1002/cam4.5953)

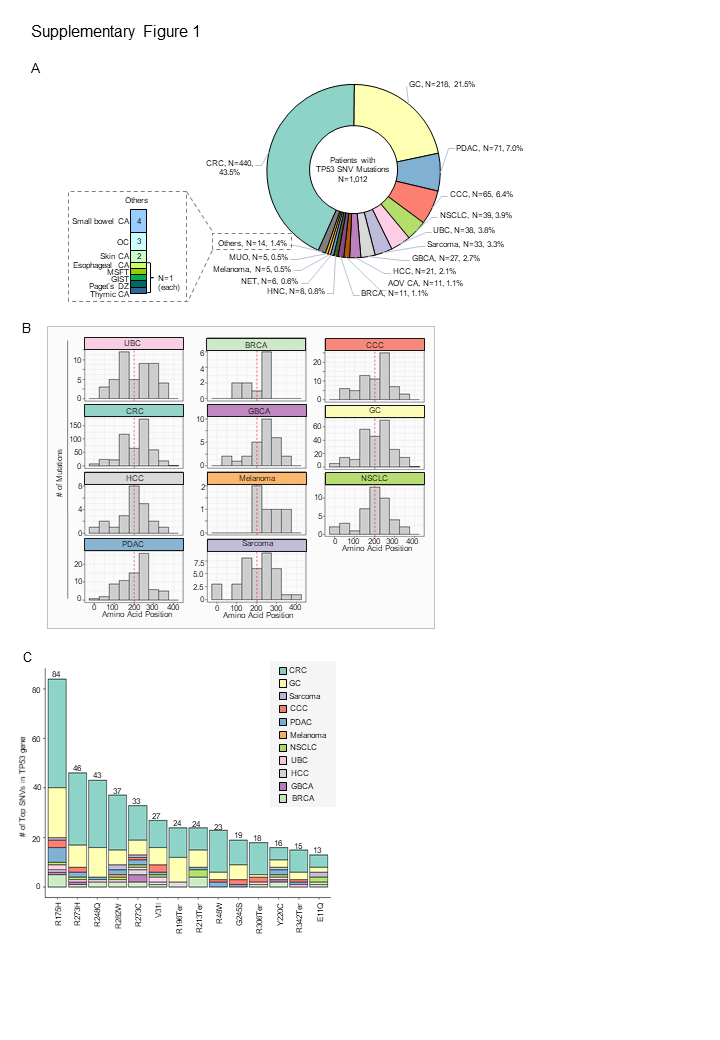

Supplement: Supplementary file 1 — Figure S1. [file CAM4-12-12438-s003.TIF]

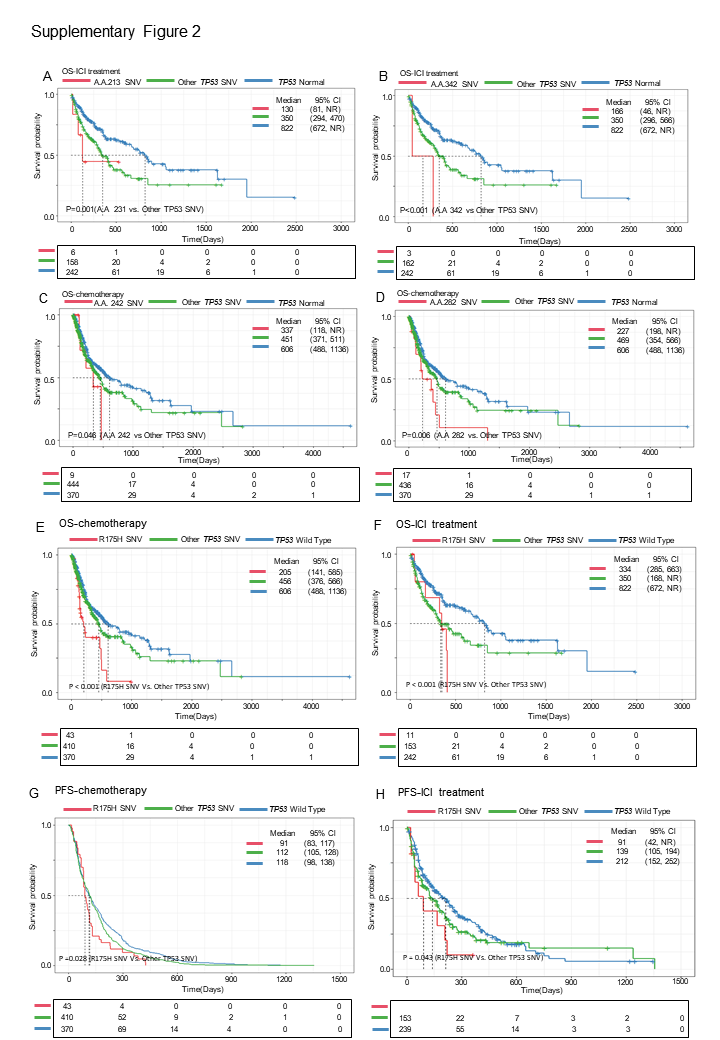

Supplement: Supplementary file 2 — Figure S2. [file CAM4-12-12438-s004.tif]

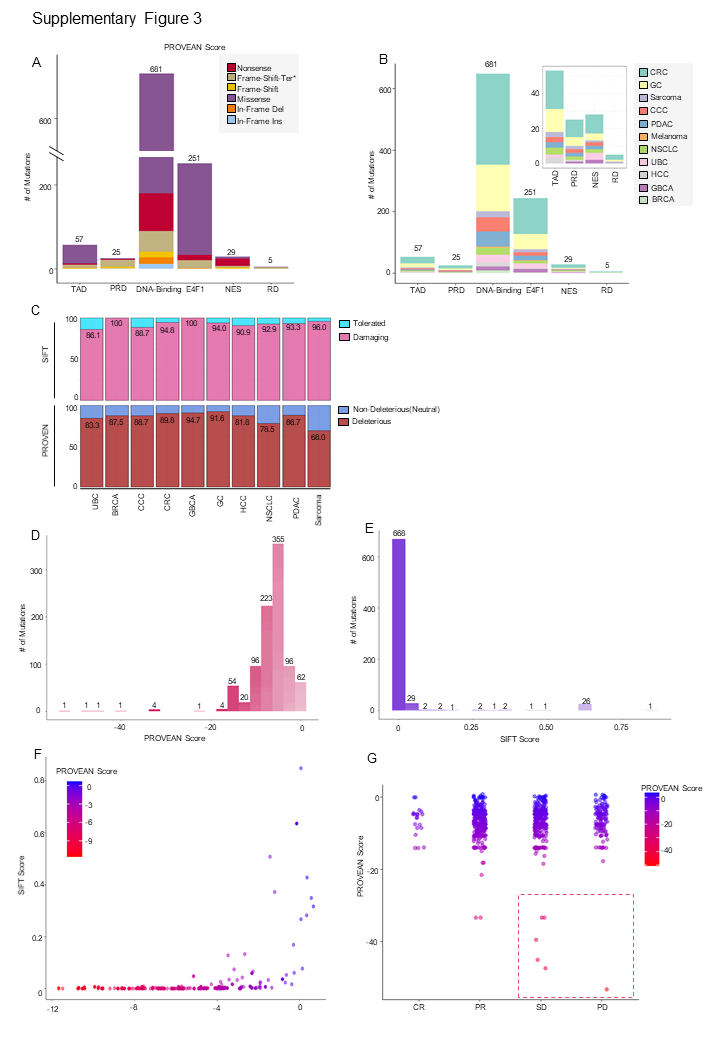

Supplement: Supplementary file 3 — Figure S3. [file CAM4-12-12438-s001.TIF]

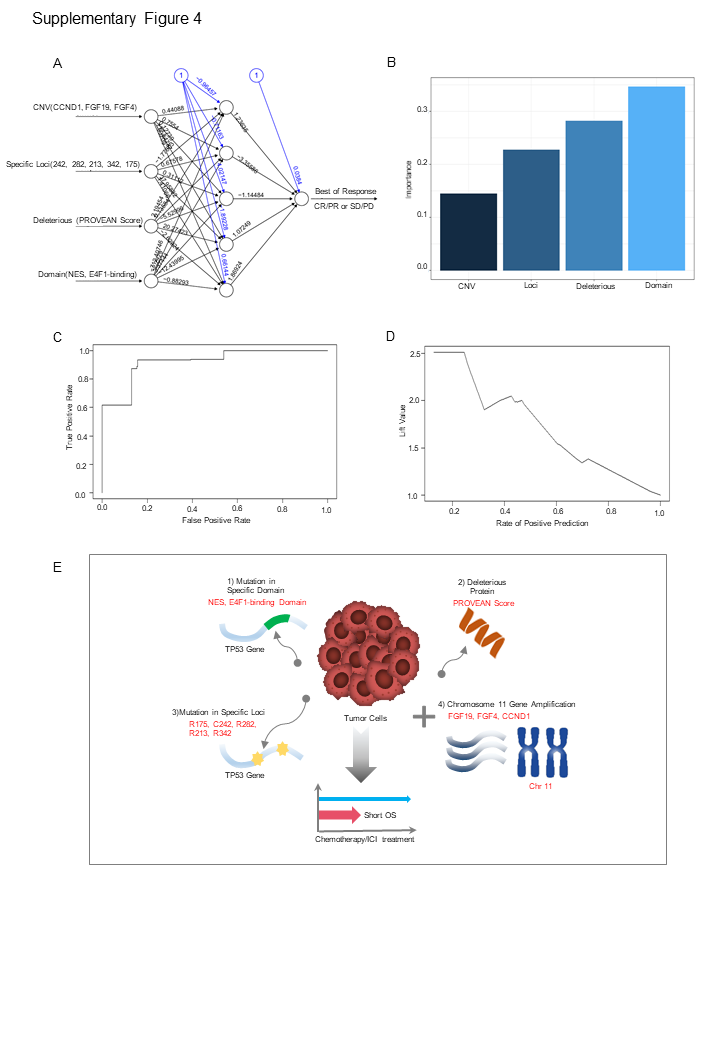

Supplement: Supplementary file 4 — Figure S4. [file CAM4-12-12438-s002.TIF]
